# Diversity, Inclusion, and Digital Preservation

**DOI:** 10.1016/j.patter.2020.100152

**Published:** 2020-11-04

**Authors:** Daniel Steinmeier

**Affiliations:** 1 National Library of the Netherlands, the Hague, the Netherlands

## Abstract

We have learned from the debate on diversity and inclusion that archiving is not neutral or unbiased even though it is presented in this way. Seen from the perspective of cultural humility, we need to keep learning and challenge power imbalances from both the individual and the organizational level. This article discusses what this means for digital preservation concepts.

## Main Text

### Diversity, Inclusion, and Digital Preservation

Diversity, inclusion, and digital preservation? The title itself may give people pause to think why it would be necessary to connect a topic like inclusion with digital preservation. Isn’t digital preservation something generic that simply works for anybody involved in curating digital content for the long term? Aren’t models about digital preservation specifically designed to work for all sorts of content and all sorts of institutions? So why bother looking at this topic from the perspective of diversity and inclusion?

Undeniably, the current debate, sparked by the movement Black Lives Matter, has put this topic right back into the spotlight. Currently within the domain of digital archiving, we can see some tentative initiatives that have been directly inspired by this movement to address the topics of diversity and inclusion. At the National Library of the Netherlands, where I work, an explicit statement has been released voicing support for this movement, and research is being done on bias within artificial intelligence. However, the attention for this topic and the different insights that resulted in these initiatives are not new. Scientists have been continuously working on this topic for decades: shedding light on bias, the “invisible” effect of existing power structures, and ways of giving underrepresented groups a voice within the world of digital heritage as this can be found in the collections of libraries, archives, and museums.

The scope of this debate is enormous, and to select just a few things to take away from it is difficult. Within the scope of this article I have selected two very important points to serve as a start for talking about digital archiving and diversity. I will focus on why change is needed and what this implies for key concepts within digital preservation.

### The Need for Change

The first thing to accept is that nothing is neutral: not the technology we use, not the collections we curate, and not the metadata we create. The whole process of archiving from acquisition to access is bound by policies, conventions, and—sometimes unwritten—rules that determine how we handle digital material but also frame our way of thinking about the material itself and the processes needed for curation. These conventions and policies originated in colonial times and were often created to serve political goals. Without investigation, the implications of these origins and political conventions are in practice invisible to us, and we are misled into thinking archiving is neutral, also because it has always been presented in this way.^1^

Only when thinking about alternatives do we start to realize the subjectivity encoded within archival practice. We should not aim for more objectivity but rather try to expose the subjectivity and find a way to accommodate views from different perspectives. In this way our policies and practices are opened for critical analysis, and improvements can be realized. This should happen in contact with the community. The goal is to create a reciprocal relationship with diverse communities so as to provide archives with a channel through which alternative views and ideas may flow. These can then be used for exposing the subjectivity of things formerly considered neutral, thus paving the way for more inclusive processes, collections, and descriptions.

### Backlash and Face-Saving

While this approach to inclusion may sound completely reasonable, many people completely misunderstand even the basic idea of this undertaking by assuming that taking on this challenge will mean that any material not reflecting contemporary ideas on diversity and inclusion will be eliminated or repressed. A statement voicing support for diversity could thus easily lead to accusations of book burning or wanting to “change history.” Bound up with these sentiments is the fact that working in cultural heritage, for instance within an archive or a library, in society at large is considered to be something beyond reproach, almost like a vocation in a religious sense.^2^ This makes it harder for employees to effect change from within. After all, advocating for change means something is not as it should be, and especially in regard to this topic, implicit racism is not something people want to own up to. Therefore, bringing up this subject may result in face-saving reactions and microaggressions meant to devalue or minimize the concerns raised. These types of reactions can be expected both within the organization and outside of it, on social media, and in contact with users. It is therefore important to present this topic in a way that may preempt these defense mechanisms and provide a framework that can be used to communicate what is at stake here and what the goal should be.

### Cultural Humility

Very useful in that regard is the concept of “cultural humility.” This concept consists of three dimensions: life-long learning and self-reflection on a personal level, accountability on the organizational level, and the commitment to recognize and challenge power imbalances.^3^ The first point is grounded in the realization that diversity is not something you can achieve in a complete and definitive way. It is therefore more important we have the readiness to commit to life-long learning and not see this as something like a competency. Achieving diversity or having a completely inclusive collection should therefore not be seen as a goal, because we need to be humble enough to realize diversity is too large a subject to completely comprehend as a person or as an organization. Also, ideas are constantly changing to reflect what is happening in society, so the topic is also too changeable to ever be something that can be permanently achieved. The goal should be to improve ourselves constantly and be transparent about this, not to strive for an “ideal” situation that would be considered “final.”

One of the other benefits of using this model is that it takes into account both the level of the individual as well as that of the organization. It is important that this topic is not only addressed on personal initiative but that the whole organization takes responsibility.

All of this is then seen from the perspective that there are power imbalances, and both individuals and organizations should have the willingness to challenge these. To a large extent, this happens by letting go of the typical role of “expert” so a reciprocal relation can be established with communities.^3^

Also within an organization, it is important that power structures are perceived and the effect of existing hierarchies is foregrounded. Subject matter experts need to be able to freely exchange ideas and set up grassroots initiatives that may provide insights. It is important to establish a safe environment for free thinking within the organization and in contact with other organizations without being restrained in advance by typical management concerns such as planning, roles, prioritization, or budgets. This is something that requires special attention since diversity initiatives often function within existing project structures and organization hierarchies that may limit out-of-the-box ideas. Management should provide support, but it is important that ideas can be formed in a reciprocal form of contact between institutions and communities without the institution determining in advance the limits of this working relationship. Communities should feel they have *agency* in the sense that they are in the lead when it comes to specifying their needs and determining what activities would fit to fulfill these needs.

### The Link with Digital Preservation

So what does all of this have to do with digital preservation? I think many concepts and models current in digital preservation could be used to challenge power imbalances on the organizational level. If we decide to look at digital preservation concepts from the perspective of diversity, they may provide a mechanism and a structure to guide self-improvement.

When we talk about digital preservation as a model, we mean first and foremost the open archival information system (OAIS) model.^4^ This is a functional model that describes all the processes a digital repository needs to implement to be able to preserve digital objects for the long term. The whole life cycle of the object from ingest to access is modeled in groups of functions called functional entities. Also part of the model are the stakeholders of the different processes, like the producers that deliver material and the designated community that makes use of the material by searching, accessing, and using the content.

Related to the functional model are a set of guidelines standardized as ISO-16363.^5^ This standard outlines the requirements an organization needs to implement to be considered a trustworthy digital repository in a formal certification procedure. It also details the evidence needed to prove said requirements are implemented as specified.

### Designated Community

The first example of how digital preservation concepts could relate to diversity revolves around the concept of the “designated community”: within the OAIS model, the designated community is an abstraction of all the different user groups a digital repository has. These various groups can be distinguished based on the specific needs of the group and the services the repository provides specifically for this group. All the subgroups in total make up what is called the designated community. It provides a reference to archives for verifying the effectivity of its policies.

One requirement states, for example, that a trustworthy digital repository must ensure that digital material is understandable to the designated community. In practice this might mean among other things that there must be enough metadata for users to be able to find it. The repository must have a mechanism to check with the relevant user group whether this is indeed the case and, if not, improve it. It should also keep track of all the changes and verify whether the measures for improvement really were effective. When thinking about archival description and inclusion, it is important to consider that the debate is not only about using words that reflect current thinking on what are appropriate terms but also about usability: are people able to search in their own language, are things described as people would describe it themselves? If not, chances are that, in practice, material is not considered findable by the designated community, and the repository should take steps to improve this.

**Figure 1:**
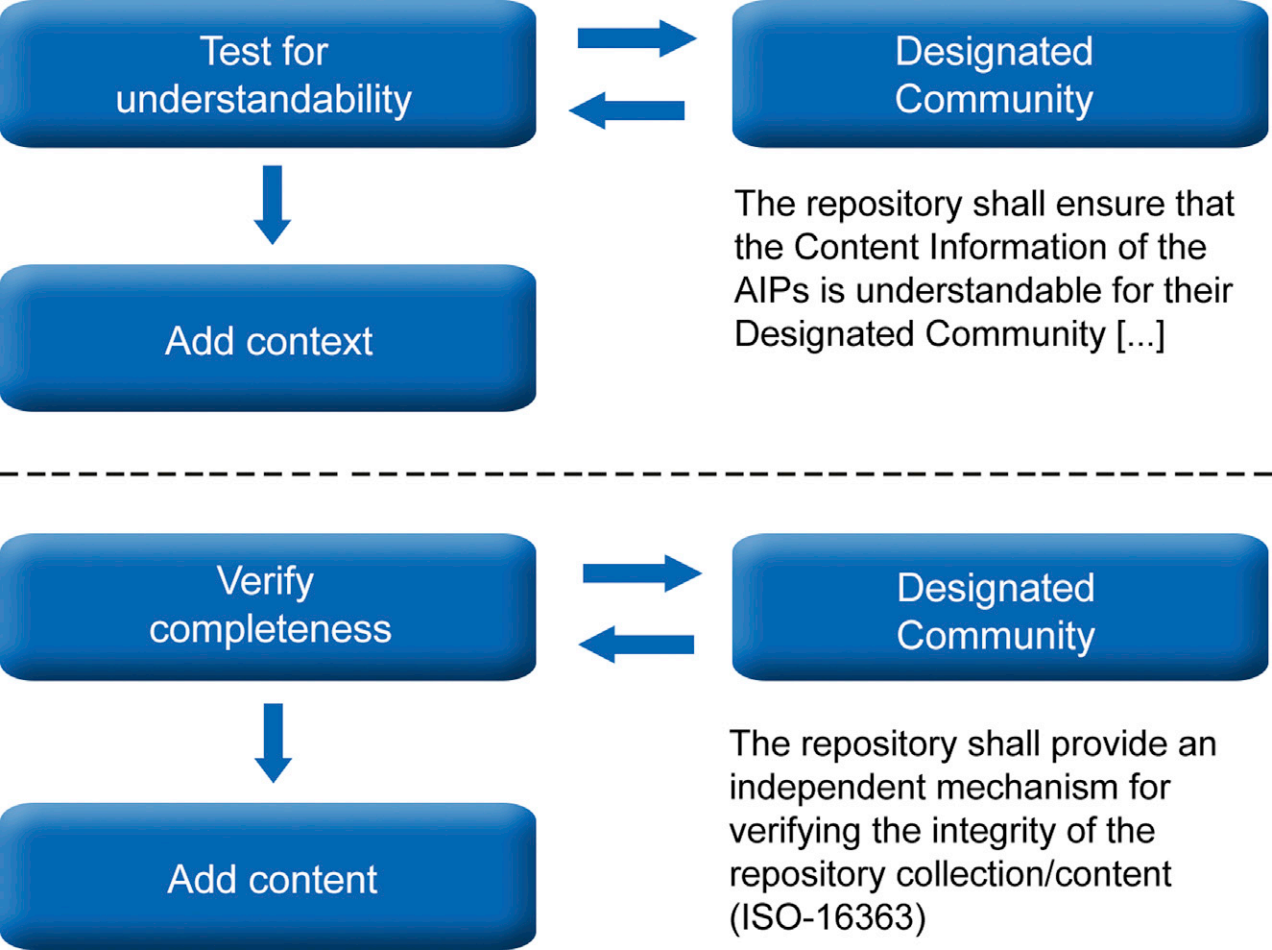
Using Feedback from the Designated Community to Improve Understandability and Completeness

This whole process of improvement based on user research can also be very relevant when viewed from the perspective of diversity. If repositories take care that the subgroups defined within their designated community also include underrepresented groups, then the processes needed to give a voice to these groups and improve things based on their feedback completely fits within the existing model. The same would go not only for metadata describing objects but also for the acquisition process itself. The repository should have an independent mechanism for determining completeness of the collection: a community could serve as a body for determining completeness or providing information for detecting gaps (Figure 1). In this way, a form of community-based acquisition could be realized in line with the ideas of people advocating for this from the viewpoint of challenging power imbalances.^6^

### Provenance

But we should not make the same mistake of thinking digital preservation itself is neutral—some concepts may turn out to be restrictive or biased in practice and might need changing. Also, some concepts may need to be implemented in a way that runs counter to current practice in order to accommodate multiple perspectives. A case in point would be the concept of provenance.

Say I wanted to tell people a personal story about my career but the only means I had of telling this story were official documents sent to me by my different employers. From this, people would get a glimpse of when my function changed or when I switched jobs, but they could never grasp what motivations and actions were behind this, let alone the emotional side of it that might be very important from the perspective of telling a personal story. As a person I would be next to invisible—just a name with some milestones and date stamps. As a story it would be very elliptical, formal, and one sided.

The provenance of the documents, the whole history starting with its creation, would be constructed from the viewpoint of the institutions that created the document, not from the person receiving it, let alone the whole context that led to the situation described in the document as an end result.

In this way, underrepresented groups can be made invisible in the archival record because they are only spoken about by others. The stories that they would like to tell about themselves cannot be heard unless we try to expand certain concepts like provenance to also include the stories of the subjects, not only those of the creators. We should investigate what the needs are of our different user groups, our designated community, and see what needs to be improved to keep our material usable and understandable for the long term. This could mean adding more context from the perspective of underrepresented groups.

### Significant Properties

The final example I would like to present deals with the concept of “significant properties.” This concept has been a complicated and much-discussed topic for years within the digital preservation community. In its most basic form, it could be explained as this: file formats may become obsolete in the future, so to preserve them a repository might transform the old format to a newer, better supported format. This process might result in the loss of some data, so the repository should specify which parts of the data are considered “significant” and make sure at least these data are preserved in the new format. This implies that some data that exist in the original format are in a way expendable. In this way it immediately becomes clear that, while at first glance this might seem a completely neutral technical choice, actually a power imbalance is visible here. After all, who gets to decide what is significant or not? And significant to whom? By making this choice, we give priority to the needs of some communities at the cost of others, effectively in the same way as is the case in an appraisal process.^7^

If we decide some features of the original object are not used by the majority, we might deem them insignificant for that reason, and this is precisely where underrepresented groups may be impacted because they might not have the numbers behind them.

### Conclusion

In the end, every process that implicitly or explicitly involves selection or choice from a variety of options, excluding something is inevitable. However, we can pay attention not only to the things excluded and the why of it but also think about *who* is making the choice and most of all *who* is impacted by the decision. This will help us establish whether choices are made to reinforce or challenge existing power imbalances. In line with the concept of cultural humility, I want to stress that I am not saying digital preservation concepts are the solution for addressing diversity and inclusion. I only hope to give some starting points on how these concepts could be put to use if we look at them from the perspective of cultural humility. This might also mean using them in a completely different way then we have been used to.
